# Role of adiponectin and proinflammatory gene expression in adipose tissue chronic inflammation in women with metabolic syndrome

**DOI:** 10.1186/1758-5996-6-137

**Published:** 2014-12-09

**Authors:** Larisa Litvinova, Dmitriy Atochin, Mariia Vasilenko, Nikolai Fattakhov, Pavel Zatolokin, Igor Vaysbeyn, Elena Kirienkova

**Affiliations:** Innovation Park, Immanuel Kant Baltic Federal University, Building 3, Botkina St, Kaliningrad, Russia; Cardiovascular Research Center, Massachusetts General Hospital, 149 East, 13th street, Charlestown, MA 02129 USA; Department of Reconstructive and Endoscopic Surgery, Kaliningrad Regional Hospital, Building 74, Klinicheskaya St, Kaliningrad, Russia; Department of General Surgery, Kaliningrad Regional Hospital, Building 74, Klinicheskaya St, Kaliningrad, Russia

**Keywords:** Metabolic syndrome, Adipose tissue, Inflammation, Obesity, Adipokine, Gene expression

## Abstract

**Background:**

The purpose of this research was to study the gene expression of interleukin-6 (IL-6), tumor necrosis factor-α (TNF-α), nuclear factor kappa B (NF-κB), vascular endothelial growth factor A (VEGF-A) and adiponectin (AdipoQ) genes in the visceral (omental, mesenteric) and subcutaneous adipose tissue depots in metabolic syndrome (MS).

We studied 23 women with MS, with a mean age of 50.7 ± 4.5 years and mean body mass index (BMI) of 45.6 ± 9.8 kg/m^2^. The control group included 10 women, with a mean age of 40.6 ± 8.7 years and normal BMI (22.3 ± 3.7 kg/m^2^). The gene expression levels in the omental (OAT), mesenteric (MAT) and subcutaneous (SAT) adipose tissues were assessed by quantitative real-time PCR.

**Findings:**

Increased gene expression levels of IL-6 and TNF-α were detected in MAT in patients with MS, compared with the control group (p < 0.05 and p < 0.005, respectively). Significant positive correlations were observed between IL-6 mRNA expression levels in OAT and the content of CD14 + cells in the peripheral blood (r = 0.55, p < 0.05), as well as between NF-κB and VEGF-A mRNA levels in OAT (r = 0.43, p < 0.05) in patients with MS. The AdipoQ gene expression levels in OAT were significantly decreased in women with MS compared with the control group (p < 0.05). In addition, there were inverse correlations between AdipoQ gene levels in MAT and serum CRP levels (r = −0.63, p < 0.05), as well as between AdipoQ gene levels in MAT and serum IL-6 levels (r = −0.46, p < 0.05).

**Conclusion:**

These data demonstrate that proinflammatory gene expression of MAT in women with MS was increased compared with the control group. The AdipoQ gene expression levels in OAT were significantly decreased in women with MS compared with the control group.

## Introduction

Significant differences exist between fat depots in relation to the complications associated with MS. MS is a risk factor for systemic inflammation, cardiovascular diseases and thrombosis [1], and it has been associated with increased levels of visceral fat, insulin resistance, arterial hypertension, lipid and carbohydrate metabolism disorders [2]. Approximately 20% of people in the general population have MS, of whom 50% have abnormal glucose tolerance and more than 80% have diabetes [3].

Adipose tissue produces adipokines, hormones and cytokines and contains various cells, such as adipocytes, preadipocytes, vascular cells, endotheliocytes, lymphocytes and macrophages expressing CD14 receptors [4–7]. In obesity, the increased production of inflammation mediators in the adipose tissue, liver, pancreas and skeletal muscles causes subclinical metabolic inflammation [5, 8, 9]. This inflammation affects the metabolic and secretory function of adipose tissue and plays a leading role in the development of obesity accompanying MS, type 2 diabetes mellitus and atherosclerosis [10–12]. The main source of proinflammatory mediators in this process are CD14 + cells [11]. Furthermore, metabolic inflammation also involves cell infiltration, fibrosis development, impaired microcirculation, enhanced adipokines and proinflammatory cytokines in the VAT (visceral adipose tissue) and increased levels of non-specific inflammatory biomarkers, such as CRP (C-reactive protein), fibrinogen, and leukocytes in the blood [13–15]. The level of inflammatory response strongly correlates with the degree of obesity [16].

Visceral and subcutaneous adipocytes might have different properties in the production of bioactive molecules. A high rate of IL-6 production in OAT suggests that IL-6 is an important regulator of adipose tissue metabolism. The gene expression profiles are different between visceral and subcutaneous fat depots [17]. However, in other studies, TNF-α mRNA expression levels were shown to be similar in these two sites [18].

There are also sex differences in fat distribution between men and women that may contribute to insulin resistance and obesity-related metabolic abnormalities [19]. Men accumulate more fat in the central (abdominal) area (mostly in visceral depots), while overweight women have demonstrated a tendency to accumulate subcutaneous fat [20]. Despres et al. showed a positive correlation between blood glucose levels and visceral adipose tissue in obese women [21]. Moreover, in addition to sex-specific patterns, age may play an important role in risk of MS and its complications [22].

It is worth noting that the visceral fat is composed of omental and, to a lesser degree, mesenteric adipose tissue. The physiological differences in the metabolism and gene expression patterns between MAT and OAT are still unknown. Accordingly, the detailed study of adipose tissue-specific proinflammatory cytokines and AdipoQ gene expression profiles is important for the estimation of the contributions of these two types of VAT to chronic adipose tissue inflammation in MS.

## Materials and methods

### Subjects

We studied the different adipose tissue depots taken from 33 patients during routine laparoscopic surgery.

The patients were divided into two groups according to their BMI.

The subjects (n = 23) were women with MS. The diagnosis of MS was defined according to the International Diabetes Federation (IDF) criteria (2005) [23]. The mean age of the study population was 50.7 ± 4.5 years and the mean BMI 45.6 ± 9.8 kg/m^2^. The control group included 10 women with a mean age of 40.6 ± 8.7 years and normal BMI (the mean BMI was 22.3 ± 3.7 kg/m^2^). All subjects gave informed consent, and the study was approved by the Local Ethics Committee of the Baltic Federal University.

### Blood

Blood was obtained by puncture of the cubital vein and taken in the morning after 12 hours of fasting. The blood was collected in Vacuette vacuum tubes with serum clot activator or EDTA for plasma preparation.

### Determination of CD14 + сells in peripheral blood

The determination of cells positive for the surface marker CD1 in the peripheral blood was performed by MACS Quant laser flow cytometry (Miltenyi Biotec, Germany) using monoclonal antibodies (MABs) with fluorescent labels FITC (Fluorescein isothiocyanate) and R-PE (R-Phycoerythrin) (Abcam, UK).

### Determination of the serum concentrations of IL-6, TNF-α and VEGF

The serum concentrations of IL-6, TNF-α and VEGF were determined by ELISA (Vector Best, Russia) using DS2 automated ELISA analyzer (Dynex Technologies, USA), according to the manufacturer’s instructions.

### Determination of plasma adiponectin concentration

Plasma adiponectin concentration was assessed by flow cytometry on a double beam laser automated analyser (Bio-Plex Protein Assay System, Bio-Rad, USA) using commercial test kits (Bio-PlexPro Human Diabetes Adipsin and Adiponectin Assays, Bio-Rad, USA), according to the manufacturer's protocol.

### Determination of C-reactive protein in serum

The serum concentration of CRP was determined using a reagent kit (CRP FS, DiaSys, Germany).

### Tissue

OAT, MAT and SAT were obtained during laparoscopic surgery. The tissue samples were placed into RNAlater RNA Stabilisation Reagent (QIAGEN, Germany), according to the manufacturer’s protocol, to prevent RNA degradation. Total RNA isolation was performed using the ExtractRNA reagent kit (Eurogene, Russia). The quantitative determination of RNA concentration was performed using a NanoVue Plus spectrophotometer (GE Healthcare, USA). The reverse transcription of total RNA samples was performed using MMLV RT (Moloney Murine Leukemia Virus Reverse Transcriptase) reagent kit (Eurogene, Russia), according to the manufacturer’s protocol. OligodT10–primer, 20 μM, was used as a seed primer. The assessment of relative gene expression was based on real-time quantitative PCR using qPCRmix-HS SYBR reagents (Eurogene, Russia). A total of 4 μl of the complimentary DNA was used as a template.

β2-Microglobulin (B2M) was used as a normalisation gene. The following primers were used in the study: IL-6 - for 5'-cct-tcg-gtc-cag-ttg-cct-tc-3', IL-6 - rev 5'- gtg-ggg-cgg-cta-cat-ctt-tg-3'; AdipoQ for both transcript variants - for 5'-caa-cat-gcc-cat-tcg-ctt-t3', AdipoQ - rev 5'- gga-ggc-ctg-gtc-cac-att-at-3'; TNF-α - for 5'-ccc-tca-acc-tct-tct-ggc-tca-a-3', TNF-α - rev 5'-cca-ggt-ttc-gaa-gtg-gtg-gtc-t-3'; NF-κB - for 5'-gtg-gtg-cct-cac-tgc-taa-ct-3', NF-κB - rev 5'-gga-tgc-act-tca-gct-tct-gt-3'; VEGF-A - for 5'-gtt-ttg-gga-aca-ccg-aca-aac-c-3', VEGF-A - rev 5'-cgg-tgt-cct-cat-ccc-tgt-acc-t-3'; B2M - for 5'-cac-ccc-cac-tga-aaa-aga-tg-3', B2M - rev 5'- ata-tta-aaa-agc-aag-caa-gca-gaa-3'.

### PCR analysis

The PCR reaction was repeated three times using the Light Cycler 480 Real-Time PCR amplifier (Roche, Switzerland). The amplification protocol was as follows: primary denaturation – 5 minutes at 95°С; amplification cycle (х 50): denaturation – 20 sec. at 95°С; primer annealing - 30 sec. at 60°С; elongation - 60 sec. at 72°С; post-incubation - 5 minutes at 72°С. The PCR results were analysed using the Second Derivative Maximum Method.

### Statistical analysis

Statistical analysis was performed using IBM SPSS Statistics 20. The nonparametric Mann–Whitney U test was used to determine the significance of differences between independent groups. The results were considered statistically significant at the 0.05 significance level. Correlation analyses were performed by calculating the Spearman rank correlation coefficient (r) to assess the relationships between the studied parameters.

Statistical analysis of PCR data was performed using the specialised software programme REST 2009 v.2.0.13. The calculation of relative gene expression in this software is based on Pfafflʼs formula [24]. The algorithms for the statistical analysis program have been validated for application to the specific randomisation test designed to interpret the real-time quantitative PCR data. The statistical significance of the differences in expression levels between the control and studied groups was evaluated for each gene by randomisation tests and was based on the mean Cp values. Differences were considered significant at a significance level of p < 0.05.

## Results

**Figure 1:**
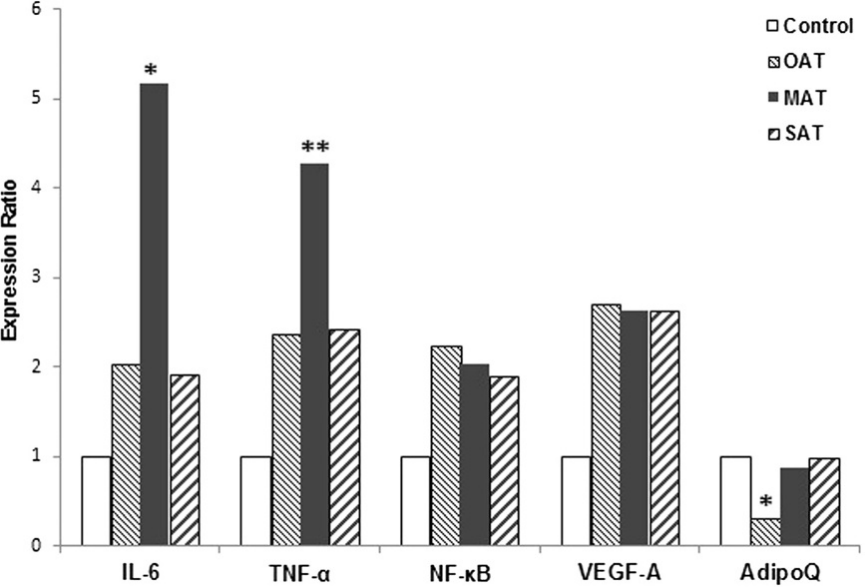
**Relative expression of 5 target mRNAs in OAT, MAT and SAT in MS (n = 23 women) vs. the control group (n = 10 women).** The expression ratio is the fold change between the sample expression and the control groups. The specific mRNAs were assessed by quantitative real-time PCR assay in adipose biopsies. The gene expression was quantified by normalization to reference gene β2-microglobulin. *P < 0.05. **P < 0.005. OAT – omental adipose tissue; MAT - mesenteric adipose tissue; SAT - subcutaneous adipose tissue.

A 3-fold decrease in AdipoQ gene expression levels in the OAT was observed in patients with MS compared with the control group (p < 0.05) (Figure 1). There were no significant differences in IL-6, TNF-α, NF-κB and VEGF-A gene expression levels in the OAT of patients with MS compared to the control group. There were no significant differences in the expression levels of the studied genes in SAT in the patients with MS compared to the control group.

The IL-6 gene expression levels were 5-fold greater in women with MS than in the control group (p < 0.05) (Figure 1). The TNF-α gene expression levels were 4-fold greater in women with MS than in the control group (p < 0.005) (Figure 1). There were no significant differences in the AdipoQ, VEGF-A and NF-κB gene expression levels in MAT in the MS group compared to the control group.

The IL-6 and TNF-α serum levels were significantly higher (4.9 (3.7-6.5) and 28.3 (15.3-43.5) pg/ml) in MS than in the control group (1.3(0.5-1.9) and 3.3 (2.7-4.5)) (p < 0.05 and p <0.05, respectively) (Table 1). Serum VEGF levels were comparable to the control group and were 180 (160–320) pg/ml (p > 0.05). The plasma adiponectin concentration was significantly lower in MS (2.0 (1.4-3.1) pg/ml) than in the control group (3.6 (2.7-4.2) pg/ml).

**Table 1:** **Serum levels of proinflammatory factors (CRP, IL-6, TNF-α, VEGF) and adiponectin plasma concentrations (Me(Q1-Q3)) in patients with MS (23 women) and the control group (10 women)**

Variable	Metabolic syndrome (n = 23)	Control group (n = 10)
IL-6 (pg/ml)	4.9 (3.74-6.55) p < 0.05	1.35 (0.55-1.95)
TNF-α (pg/ml)	28.28 (15.26-43.49) p < 0.05	3.30 (2.70-4.55)
VEGF (pg/ml)	180 (160–320) p > 0.05	172 (156–290)
Adiponectin (μg/ml)	2.0 (1.4 – 3.1) p < 0.05	3.6 (2.7–4.2)

p - significance of differences as compared to the control group.

**Figure 2:**
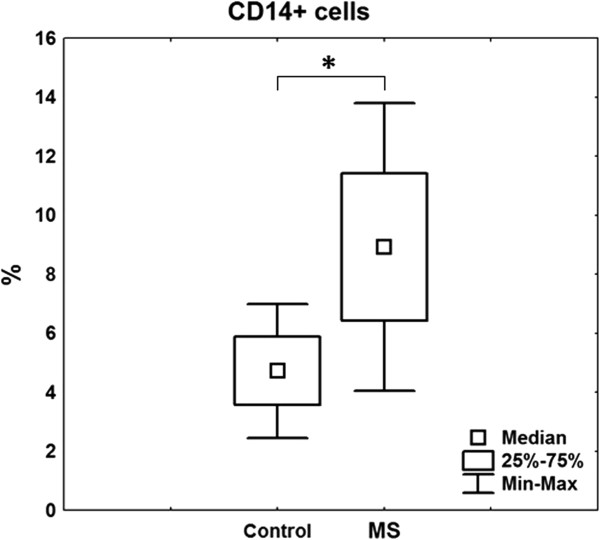
**The percentage of CD14+ сells in peripheral blood in patients with MS (23 women) and the control group (10 women).**

**Figure 3:**
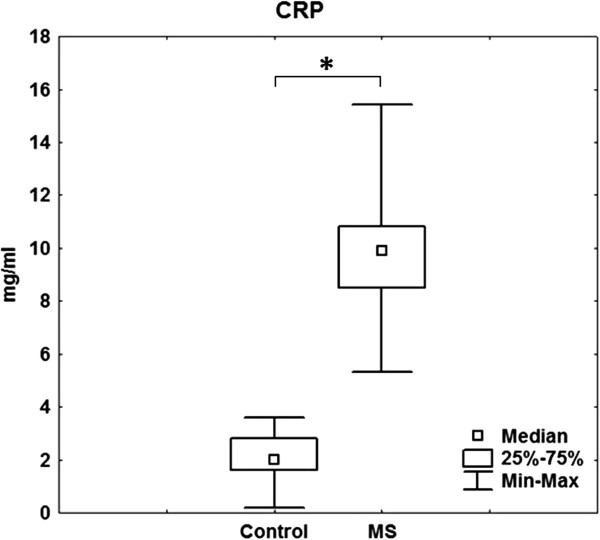
**The content of CRP in serum in patients with MS (23 women) and the control group (10 women).**

We demonstrated a significant increase in the content of CD14 + cells in the peripheral blood in patients with MS (8.9 (8.2-9.8) compared with the control group (4.8 (4.0-5.4)) (p < 0.05) (Figure 2). The serum CRP concentration in patients with MS was 10.6 (9.9 - 13.4) mg/l, exceeding the value of the control group, which was on average 5-fold (p = 0.001) (Figure 3).

**Figure 4:**
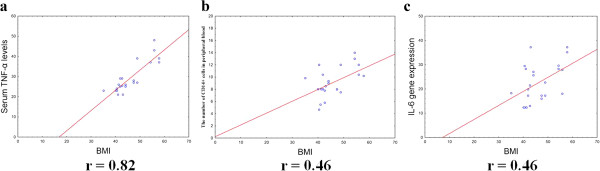
**Correlation between serum BMI and TNF-α levels (a), content of CD14 + cells in the peripheral blood (b), IL-6 gene expression in OAT (c).** The gene expression levels were assessed by quantitative real-time PCR assay in OAT from 23 women with MS and the control group (10 women). The Pearson correlation coefficients (r) are shown on the figure and the P value if statistically significant with a P < 0.05.

**Figure 5:**
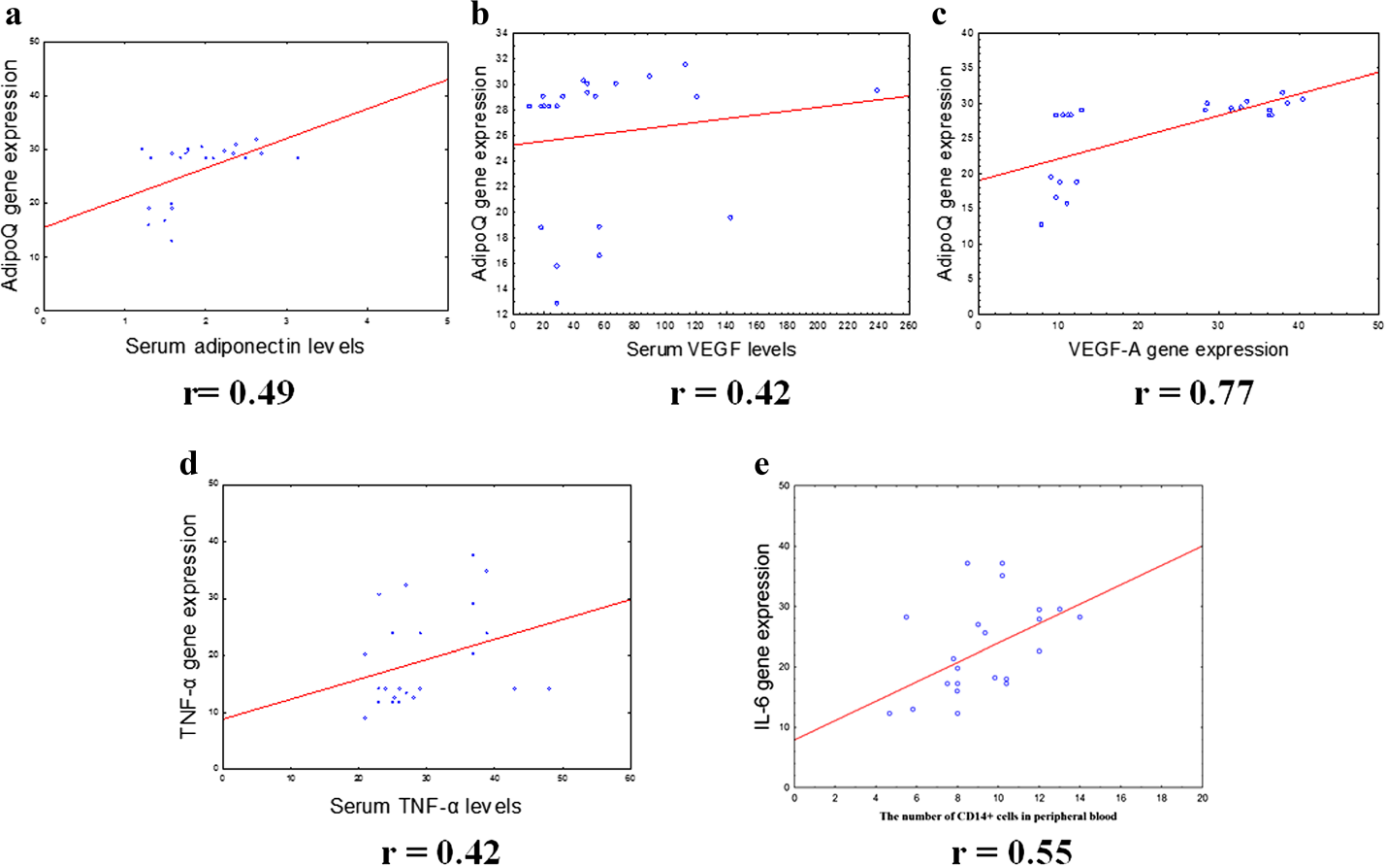
**Correlations between AdipoQ α gene expression in OAT and plasma adiponectin levels (a),serum VEGF levels (b), VEGF-A gene expression in OAT (c), between and TNF-α gene expression in OAT and serum TNF-α levels (d), IL-6 gene expression in OAT and content of CD14 + cells in the peripheral blood (e).** The gene expression levels were assessed by quantitative real-time PCR assay in OAT from 23 women with MS and the control group (10 women). The Pearson correlation coefficients (r) are shown on the figure and the P value if statistically significant with a P < 0.05.

**Figure 6:**
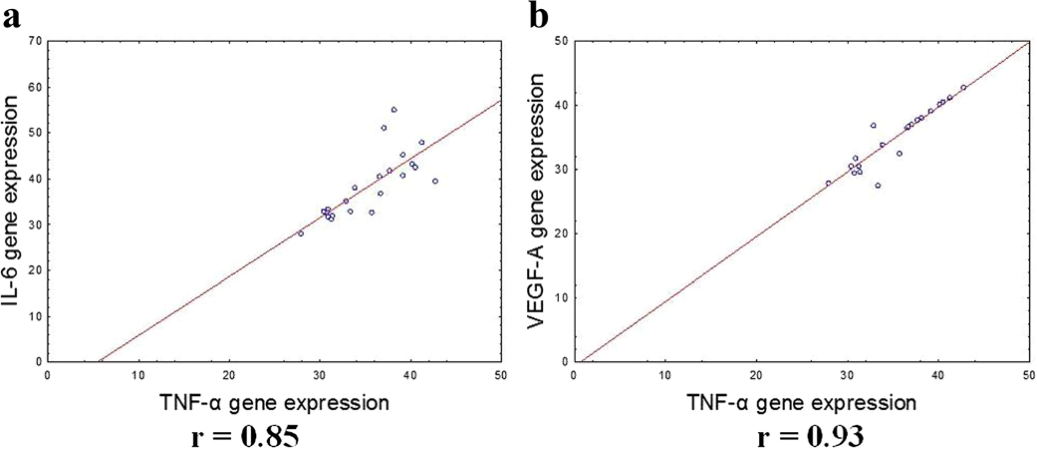
**Correlation between TNF-α gene expression in SAT and IL-6 geneexpression in SAT (a), VEGF-A gene expression in SAT (b).** The gene expression levels were assessed by quantitative real-time PCR assay in SAT from 23 women with MS and the control group (10 women). The Pearson correlation coefficients (r) are shown on the figure and the P value if statistically significant with a P < 0.05.

There were positive correlations between IL-6 gene expression levels in OAT and BMI (r = 0.46, p < 0.05) and between serum TNF-α levels and BMI in patients with MS (r = 0.82, p < 0.05) (Figure 4). The serum TNF-α levels correlated with TNF-α gene expression in OAT (r = 0.42, p <0.05) (Figure 5). Significant correlations were observed between IL-6 gene expression levels in SAT and TNF-α gene expression in SAT (r = 0.85, p < 0.05), as well as TNF-α gene expression in SAT and VEGF-A gene expression levels in SAT (r = 0.93, p < 0.05) (Figure 6).

**Figure 7:**
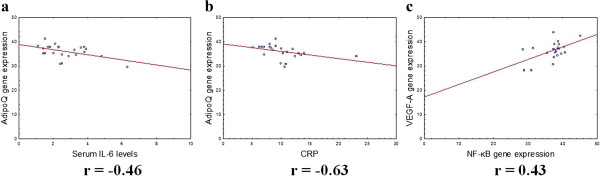
**Correlations between serum AdipoQ gene expression in MAT and serum IL-6 levels (a), serum CRP levels (b), between VEGF-A gene expression in MAT and NF-κB gene expression in MAT (c).** The gene expression levels were assessed by quantitative real-time PCR assay in MAT from 23 women with MS and the control group (10 women). The Pearson correlation coefficients (r) are shown on the figure and the P value if statistically significant with a P < 0.05.

We found that in MAT, the VEGF-A gene expression levels correlated with NF-κB gene expression levels (r = 0.43, p < 0.05) (Figure 7). Moreover, in OAT, we revealed a positive correlation between VEGF-A gene expression levels and AdipoQ mRNA expression (r = 0.77, p < 0.05), as well as serum VEGF levels and AdipoQ gene expression levels (r = 0.42, p < 0.05) (Figure 5). We also observed a positive correlation between AdipoQ gene expression levels in OAT and its plasma levels (r = 0.49, p < 0.05) (Figure 5).

We demonstrated a negative correlation between AdipoQ gene expression in MAT and serum IL-6 levels (r = −0.46, p < 0.05), which indicates the development of inflammation in MS (Figure 7). We also showed a negative correlation between the level of AdipoQ gene expression in MAT and the serum CRP levels (r = −0.63, p < 0.05) (Figure 7).

We found positive correlations between the number of CD14 + cells and IL-6 gene expression levels in OAT (r = 0.55, p < 0.05) (Figure 5). Of interest, we also demonstrated a positive correlation between the number of CD14+ cells in the peripheral blood and BMI (r = 0.46, p < 0.05) (Figure 4).

## Discussion and conclusion

The adipocytes, preadipocytes and macrophages infiltrating adipose tissue in obesity are sources of proinflammatory cytokines [10, 25, 26]. Cytokine production causes chronic inflammation [13], and the activation of proinflammatory mechanisms correlates with insulin resistance and atherosclerosis [10, 11, 27]. The VAT plays important roles in the development of type 2 diabetes, atherosclerosis and cardiovascular diseases [7, 10, 11].

VAT is, to a large extent, represented by hypertrophied adipocytes with a high degree of sensitivity to hormonal effects and is therefore more metabolically active than subcutaneous or extraperitoneal fat [28]. IL-6 and TNF-α are predictors of vascular complications of diabetes in obesity and play a leading role in atherosclerosis and the development of insulin resistance [10, 29]. Increased IL-6 gene expression in adipose tissue leads to an increase in IL-6 serum levels, which positively correlates with BMI [5, 6, 30]. The concentrations of IL-6 and TNF-α in the VAT are higher than its circulating levels [31, 32]. Our results demonstrate, for the first time, an increase in IL-6 and TNF-α gene expression, specifically in MAT and VAT, in patients with MS compared with the control group.

NF-κB is a transcription factor that influences the expression of various genes involved in immune and inflammatory responses [33]. The NF-κB signalling pathway involving IL-1β and hypoxia-inducible factor 1-alpha (HIF-1α) leads to the increased production of VEGF by different cells [34, 35]. VEGF-A is a key molecule in vasculogenesis, angiogenesis, vascular permeability and tissue remodelling [36, 37].

In adipose tissue, hypoxia plays an important role in the activation of the transcription factors NF-κB and HIF-1α in adipocytes and macrophages [36]. The proinflammatory cytokines can act as inducers of NF-κB activation in adipose tissue inflammation [34, 38]. To prove the influence of NF-κB gene expression on the increased levels of proinflammatory cytokines and NF-κB activation in adipose tissue, we will need to perform immunohistochemistry analysis of NF-κB nuclear localisation on tissue sections in the future.

The trend of increased VEGF-A gene expression in OAT in patients with MS compared with the control group might be associated with the activation of the NF-κB signalling pathway [26]. The overexpression of the VEGF-A gene has a protective effect against adipose tissue inflammation by attracting M2-macrophages to the fat depot, thereby maintaining the inflammatory microenvironment [39].

The literature relating to serum VEGF concentrations and its gene expression levels in adipose tissue in obesity are contradictory [40–42]. Some studies have shown that the VEGF serum levels were significantly correlated with BMI and depended on its gene expression in VAT [40, 41]. However, other authors have reported decreased VEGF-A gene expression in adipose tissue in obesity [42]. The decrease in VEGF-A production in adipose tissue in patients with type 2 diabetes may indicate a protective role of VEGF-A against insulin resistance induced by obesity [38, 39, 42, 43].

Adiponectin is a key mediator synthesised in adipose tissue. Adiponectin improves insulin sensitivity and enhances insulin effects in peripheral tissues, particularly in the muscle, liver and adipose tissues. Decreased secretion of adiponectin in obesity is one of the leading factors involved in the development of insulin resistance [44, 45].

We demonstrated significant changes in AdipoQ gene expression levels in OAT in patients with MS compared with the control group. According to previous studies, a higher level of secretion was observed in SAT than in VAT [17, 45]. Another study suggested that AdipoQ gene expression in SAT exhibited an inverse correlation with plasma adiponectin levels [46]. The decrease of plasma adiponectin levels in patients with MS confirmed the data in the existing literature [17, 47].

The expression of CD14 in macrophages and monocytes participates in activation via a mechanism mediated by Toll-like receptor-4 and a receptor for complexes of lipopolysaccharide (LPS) and LPS-binding protein [48, 49]. The presence of positive correlations between the number of CD14 + cells and IL-6 gene expression levels in OAT reflects the fact that an increase in blood CD14 + cells is mediated by the increased production of proinflammatory cytokines in adipose tissue [10, 11].

Serum CRP level is an integral marker of metabolic disorders [50]. In our study, serum CRP levels were increased in patients with MS compared with the control group. According to the literature, elevated CRP serum levels in patients with MS were mediated by the ability of adipose tissue to maintain high-levels of synthesis of inflammatory mediators (IL-6, TNF-α and PAI-1) [51], which can stimulate CRP production by liver cells [52].

In fact, in a number of studies, adiponectin concentrations have been found to be inversely associated with systemic inflammation and increased concentrations of high-sensitive C-reactive protein (hs-CRP) [53]. Previous studies have indicated that plasma hs-CRP levels were negatively correlated with plasma adiponectin levels in patients with coronary artery disease [53]. The adiponectin/CRP ratio was significantly lower in MS subjects than in control subjects in another study [54]. It has been previously shown that adiponectin reduces CRP synthesis and secretion from human aortic endothelial cells under hyperglycaemia via the upregulation of AMP kinase and the downregulation of NF-κB [55].

The inverse correlation between MAT AdipoQ gene expression and serum CRP levels may indicate the initiation of proinflammatory processes in MAT under the influence of CRP in MS and obesity-related diseases. Interestingly, this significant inverse correlation was observed between CRP and adiponectin mRNA levels in human SAT [53].

IL-6 is one of the inducers of CRP synthesis and secretion by adipocytes in MS [56]. It also explains the negative correlation between MAT AdipoQ gene expression and serum IL-6 levels in women with MS.

It has been concluded in previous studies that the mesenteric adipose tissue has the highest levels of gene expression [57]. Our study identified a significant increase in IL-6 and TNF-α gene expression levels in the mesenteric adipose tissue and indicates an enhanced proinflammatory process in MS. Accordingly, a recent study has demonstrated a direct relationship between the thickness of the mesenteric adipose tissue and intima-media thickness [58]. Our results confirmed the data obtained in a study of mesenteric fat thickness, indicating the dependence of the risk of MS on the amount of MAT [59].

Thus, we have established that the proinflammatory properties of adipose tissue depend on its localisation in women. We have showed a significant increase in the gene expression levels of IL-6 and TNF-α in MAT and a decrease in AdipoQ gene expression levels in OAT in women with MS compared with the control group. These results allow us to conclude that VAT is more involved in chronic adipose tissue inflammation than SAT. We demonstrated that MAT is more inflammatory than OAT and SAT in women with MS. The interrelation with key proinflammatory serum markers, namely, the NF-κB and VEGF-A mRNA levels, confirm that all three types of fat depots participate in systemic inflammation in women with MS. Understanding the molecular mechanisms of energy homeostasis disorders will allow personalised targeted therapy based on the molecular signature of inflammatory markers of the adipose tissue.

## Authors’ information

Larisa Litvinova – MD, PhD, Head of Laboratory immunology and cell biotechnology, Innovation Park, Immanuel Kant Baltic Federal University. Atochin Dmitriy – MD, PhD, Assistant Professor of Medicine, Cardiovascular Research Center, Massachusetts General Hospital, Harvard Medical School. Mariia Vasilenko – PhD-student Laboratory of Immunology and Cell Biotechnologies Immanuel Kant Baltic Federal University. Nikolai Fattakhov – PhD-student Laboratory of Immunology and Cell Biotechnologies Immanuel Kant Baltic Federal University. Pavel Zatolokin – MD, Head of Department of Reconstructive and Endoscopic Surgery, Kaliningrad Regional Hospital. Igor Vaysbeyn - MD, Head of Department of General Surgery, Kaliningrad Regional Hospital. Elena Kirienkova – MD, PhD, Laboratory of Immunology and Cell Biotechnology, Innovation Park, Immanuel Kant Baltic Federal University.
